# Reference Depolarization Values for Polar-Organic Aggregates

**DOI:** 10.1021/acs.jctc.5c01055

**Published:** 2025-10-27

**Authors:** Gabriela Herrero-Saboya, Matic Poberznik, Nicolas Salles, Layla Martin-Samos

**Affiliations:** † 518735 CNR-Istituto Officina Dei Materiali (IOM), C/O SISSA, Trieste I-34136, Italy; ‡ Department of Physical and Organic Chemistry, 61790 Jožef Stefan Institute, Jamova 39, Ljubljana SI-1000, Slovenia

## Abstract

A key aspect in the design of mixed polar-organic layers and metallic or semiconducting devices is the dielectric constant of the organic aggregate, which modulates the surface work function and band alignments. In simple electrostatic models, a monolayer is treated as an array of point dipoles whose depolarization depends only on an effective molecular polarizability. However, the absence of a unified framework in quantum-chemical computational studies leaves the reliability of the point dipole model uncertain. In this work, we demonstrate the breakdown of the point dipole approximation for highly packed aggregates and propose an alternative heuristic model that relies on a second molecular parameter: the molecular size. The performance of this approach is validated through comparison with computational methods based on Density Functional Theory (DFT) and second-order Møller–Plesset perturbation theory (MP2) calculations. Our extended dipole model provides robust estimates of depolarization effects, offering a rapid prescreening tool for selecting polar organic candidates in surface functionalization.

## Graphical abstract

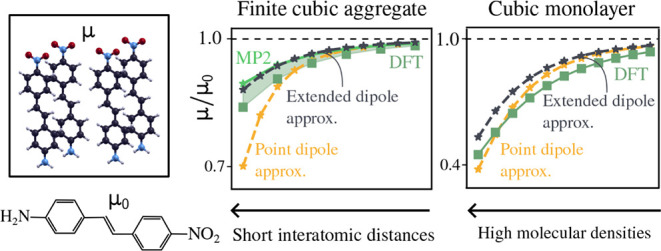

## Introduction

Polar organic aggregates-particularly self-assembled monolayers (SAMs)-have been extensively employed to functionalize metallic and semiconducting surfaces, with early foundational studies by I.H. Campbell et al.^1,2^ These systems enable control over charge injection processes by modifying the interfacial energetics through molecular dipole engineering. Although the design of such hybrid organic/inorganic interfaces is conceptually straightforward, the collective behavior of molecular aggregates often deviates significantly from that of their isolated counterparts in the gas phase. Notably, the ratio between the molecular dipole moment in the gas phase and its effective value in the condensed (aggregated) state is a key parameter in determining the dielectric properties of the monolayer, specifically its effective dielectric constant or apparent relative permittivity. Accurately determining the relative permittivity is crucial for prescreening polar ligand candidates in interfacial engineering, as variations in work function and band alignment are proportional to this parameter.

The depolarization of a monolayer can be qualitatively understood through simple electrostatic considerations, modeling an aggregate of polar ligands as an infinite array of point dipoles and estimating the reduction in dipole moment caused by the electric fields of neighboring dipoles (see for example).^3^ Within the point dipole approximation (PDA), the dielectric constant of the monolayer depends on the lattice symmetry, the molecular density and a single molecular parameter: an effective molecular polarizability. Although a simplistic approach, this classical model has served as reference for studies exploring depolarization effects in a wide range of polar-organic aggregates.

While computational studies have investigated the influence of specific polar-organic molecules on selected substrates-such as halide perovskites for photovoltaic applications^4^ and metals or transition metal oxides for rechargeable battery systems^5–7^ -quantum-chemical investigations focusing solely on depolarization effects within polar-ligand aggregates remain relatively limited.^8–11^ Existing studies have primarily focused on the phenomenological description of these effects, rather than on the development of a comprehensive theoretical framework capable of capturing the underlying mechanisms/behavior across different systems. Early Density Functional Theory (DFT) calculations revealed that the reduction of dipole moment at a semiconductor-monolayer interface matches that of the *isolated monolayer*, due to intramolecular charge rearrangements.^8^ Surprisingly, few estimated dipoles in the monolayer had a greater magnitude than that in the gas-phase, μ_0_, even for nonpolar benzene rings. Semiempirical calculations later related the depolarization in finite clusters to aggregate size, π-conjugation length, and packing motif.^9^ A benchmark study on monolayers compared the Perdew–Burke–Ernzerhof (PBE) exchange-correlation functional and hybrid/orbital-dependent functionals against coupled-cluster theory.^10^ It showed *convex* depolarization curves with respect to molecular density, rather than concave and gradually converging to μ_0_ at low molecular densities. Spurious dipole–dipole interactions in periodic-cell calculations were further exposed in ref.  ^11^.

The lack of a unified framework in depolarization studies of organic aggregates leaves the accuracy of computational methods uncertain. Furthermore, while the validity of the point dipole approximation has been questioned for aggregates where the intermolecular separation is comparable to molecular size, no quantum-chemical study has directly assessed its reliability. Consequently, the conceptualization of mixed devices remains largely empirical, limiting rational design based on predictive models.

In this work, we systematically investigate depolarization effects in polar ligand aggregates, explicitly evaluating the accuracy of the classical model against computational approaches based on the PBE functional and second order Møller–Plesset perturbation theory (MP2). By examining highly packed monolayers, we observe the breakdown of the point dipole approximation and propose an alternative heuristic approach, the extended dipole approximation (EDA). The EDA delivers near-quantum accuracy and outperforms the PDA without increasing computational cost.

Our model for molecular aggregates are isolated finite and infinite square aggregates with varying intermolecular distances (or molecular densities). We focus on elucidating the dependency of the molecular dipole moment in aggregates on three main aspects: the size of the aggregate, the intermolecular distance and the nature of the compound (characterized by size, dipole moment in vacuum, molecular polarizability, etc.). From the vast catalog of polar ligands, we first select a simple liner molecule to be used as a ″toy-model″ for depolarization curves: the hydrogen cyanide (HCN). We then considered donor–acceptor compounds, for which the -NH_2_ and -NO_2_ radicals are separated by π-chains of different size.

## Electrostatic Models for Depolarization Effects

We consider square finite and infinite aggregates of polar ligands in both one and two dimensions with varying intermolecular distances. The displacement vectors are defined within the plane perpendicular to the dipole moment of the polar ligand, which is taken to be the *z*-axis. The position/displacement vectors of the molecules in two-dimensional aggregates are thus,

1 r⃗=naî+maĵ

where *a* is the fixed intermolecular distance and *n* and *m* run from 0 to *N* – 1, resulting in clusters with *N × N* molecules. Equivalent finite aggregates are defined in one dimension. Besides these finite clusters, infinite one-dimensional (1d) and two-dimensional (2d) arrays are also considered. Lattice square vectors thus simply correspond to aî and aĵ for the 2d case.

### The Point Dipole Approximation

The point dipole approximation (PDA) assumes that the electric field generated by the ligands within an infinite 2d aggregate is given by a set of point dipoles of strength μ and direction k̂. The electric field produced by a single point dipole is given by,

2 $$\mathrm{E⃗}=-\frac{\mu }{r^{3}}\mathrm{k̂}$$

where *r* or |r⃗| is the distance to the point dipole in the plane perpendicular to *k̂*. In the case of a square monolayer, characterized by an intermolecular distance, *a*, the total electric field is simply,

3 $$\mathrm{E⃗}=-\frac{\mu }{a^{3}}\overset{\infty }{\underset{n=0}{\sum }}\overset{\infty }{\underset{m=0}{\sum }}\frac{1}{{\left(n^{2}+m^{2}\right)}^{3/2}}\mathrm{k̂}$$

The magnitude of these point dipoles is however affected by the surrounding dipoles in the array. The change in the dipole moments is assumed to be a linear effect, that is modulated by an *effective* molecular polarizability, α. The depolarization of the point dipoles is therefore described by,

4 $$\mathrm{\mu ⃗}={\mathrm{\mu ⃗}}_{0}+\alpha \mathrm{E⃗}$$

where μ_0_ is the magnitude of the dipole moment of the isolated molecule and E⃗ is the electric field along the direction of the dipoles.

The dipole moment μ within a square lattice can be thus obtained from Eqs 3 and 4. The infinite sum in eq 3 is often referred to as the geometrical factor and it was estimated to be equal 9.033 for a square infinite array by Topping et al.^12^ Combining eqs 3 and 4, the normalized dipole moment for the square lattice is given by,

5 $$\frac{\mu }{\mu_{0}}={[1+\frac{9.033\alpha }{a^{3}}]}^{-1}$$

where α is given in units of Å^3^. We also remark that eq 5 can also be exploited to describe the normalized dipole moment in a 1d array. The geometrical factor in this case is simply given by the infinite sum of $1/n^{3}$ , that is equal to 2.4.

*Within the point dipole approximation, the drop of the dipole moment in a monolayer is given by one single molecular parameter: the effective molecular polarizability.*

### The Point Dipole Approximation for Finite Aggregates

Aiming at unifying the framework for depolarization effects in polar ligands aggregates, we extend the standard formulation of the point dipole approximation to finite aggregates. In these cases, the electric field experienced by each molecule varies depending on the number of its nearest neighbors. In other words, the geometrical factor for each point in the aggregate differs from that of its neighboring points. We thus estimate the electric field within these aggregates by averaging the electric fields felt by each ligand. The corresponding geometrical factor for a two-dimensional aggregate is,

6 $$f=\frac{1}{N^{2}}\overset{N-1}{\underset{n=0}{\sum }}\overset{N-1}{\underset{m=0}{\sum }}\left(\overset{N-1-n}{\underset{i=0-n}{\sum }}\overset{N-1-m}{\underset{j=0-m}{\sum }}\frac{1}{{\left(i^{2}+j^{2}\right)}^{3/2}}\right)$$

where (*i,j*) ≠ (0,0), which can be evaluated numerically for any *N*. We remark that averaging the electric fields is equivalent to averaging the dipole moments of the finite array, resulting in an averaged dipole moment per molecule.

### The Extended Dipole Approximation

Finally, we propose an alternative model to the point dipole approximation to describe depolarization effects. We approximate the polar ligands as two point charges separated by a distance *d*, which corresponds to the dipole size. Molecules in an infinite square array thus experienced the following electric field,

7 $$\mathrm{E⃗}=-\frac{\mu }{a^{3}}\overset{\infty }{\underset{n=0}{\sum }}\overset{\infty }{\underset{m=0}{\sum }}\frac{1}{{[n^{2}+m^{2}+{\left(\frac{d}{2a}\right)}^{2}]}^{3/2}}\mathrm{k̂}$$

Assuming the same dependency of μ with the electric field as in the point dipole approximation (eq 4), the normalized dipole moment is given by,

8 $$\frac{\mu }{\mu_{0}}={[1+\frac{\alpha }{a^{3}}\overset{\infty }{\underset{n=0}{\sum }}\overset{\infty }{\underset{m=0}{\sum }}\frac{1}{{[n^{2}+m^{2}+{\left(\frac{d}{2a}\right)}^{2}]}^{3/2}}]}^{-1}$$

Notice that the dependency of *a* and *d* cannot be factored out of the infinity sums and thus, they have to be numerically estimated for each pair of *a* and *d* values. For all considered molecules and intermolecular distances, these sums were evaluated for *N* = 200, which was sufficient to ensure convergence. A similar expression that took into consideration the molecular size in depolarization effects was previously proposed in an hexagonal lattice.^3^ However, the infinite array was divided into two domains: using the *exact* expression of the electric field for the nearest neighbors to the observation point, while applying the point dipole approximation elsewhere. Moreover, it was tested on relatively small ligands, such as a −C–H bond, where the model’s potential was not significant.

*Within the extended dipole approximation, the drop of the dipole moment in the aggregate is determined by two molecular parameters: the effective molecular polarizability,α, and the dipole size, d*.

## Depolarization Effects in Polar-Organic Aggregates

Dipole moment calculations for isolated molecules have been extensively performed with codes for electronic structure calculations, e.g., estimating the expectation value of the dipole operator^13^ or exploiting the dipole correction method.^14–18^ Moreover, their accuracy with respect to the chosen functional (in the case of DFT) and basis set has been investigated in different benchmarks (see for instance refs. 16–20 ). As stated above, even dipole moment calculations of finite and infinite two-dimensional aggregates have been previously performed (see for instance refs. 8–11 ).

In this work, we assess depolarization effects of square finite and infinite aggregates of hydrogen cyanide and several aromatic-based compounds. For these aggregates, we compare the electrostatic or classical models against standard DFT using the PBE functional and MP2 calculations. The computational details for polar-organic molecules and aggregates can be found in the Supporting Information. In the Supporting Information, we also include structural and electronic properties of the ligands in the gas phase, together with well-established reference values.^19–21^

We emphasize that our model of polar ligand aggregates considers idealized two-dimensional arrays in which the dipoles are perfectly aligned perpendicular to the aggregate plane. In this framework, dipole moments are evaluated only along the nonperiodic direction. In more realistic situations, however, polar ligands may not be perfectly aligned to maximize the perpendicular dipole component, and parallel contributions can become relevant. In such cases, and more generally for fully periodic systems, dipole moments can be computed within the three-dimensional DFPT formalism.^22,23^

For the electrostatic models, the effective molecular polarizability, α, is defined as the iso-value of the polarizability, whereas the dipole size, *d*, is fixed to the molecular size for the extended dipole approximation. The choice of these parameters is further discussed in the Supporting Information.

### The HCN Molecule: A Proof of Concept

We consider finite and infinite square HCN aggregates in one and two dimensions, with intermolecular distances, *a*, ranging from 2.5 Å to 12.5 Å. We perform single point PBE and MP2 calculations for all considered HCN aggregates, except for infinite arrays and the 6 × 6 aggregate, for which only PBE calculations are conducted. For each aggregate, the calculated dipole moment per molecule (μ) is normalized by the corresponding calculated dipole moment in vacuum (μ_0_). As input parameters of the point and extended dipole approximations, we use the experimental iso-value of the polarizability ^21^ α_iso_ = 2.59 Å^3^ (see Supporting Information for more details) For the extended dipole approximation, the molecular size is fixed to the distance between the hydrogen and the nitrogen atoms in the optimal gas phase configuration obtained from PBE and MP2 calculations, *d* = 2.2 Å.

In Figure 1, we present the normalized dipole moment, *μ*/*μ*_0_, as a function of the intermolecular distance for all four considered approaches. These models clearly show that the depolarization of the aggregates is strongly affected by the intermolecular distance and the number of molecules in the finite aggregates. For both one and two-dimensional cases, the depolarization increases as the intermolecular distance decreases and as the number of molecules in the aggregate increases (Figure 1). For sufficiently large distances, approximately *a* = 10Å for the considered cases, the dipole moment of the aggregates, μ, converges to μ_0_. When comparing 1d and 2d arrays, depolarization is more pronounced in the latter. At *a* = 2.5 Å, a distance comparable to the size of the HCN molecule, the dipole moment is 0.76 μ_0_ for the 1d case and 0.42 μ_0_ for the 2d square array, as obtained from both PBE and MP2 calculations.

**Figure 1:**
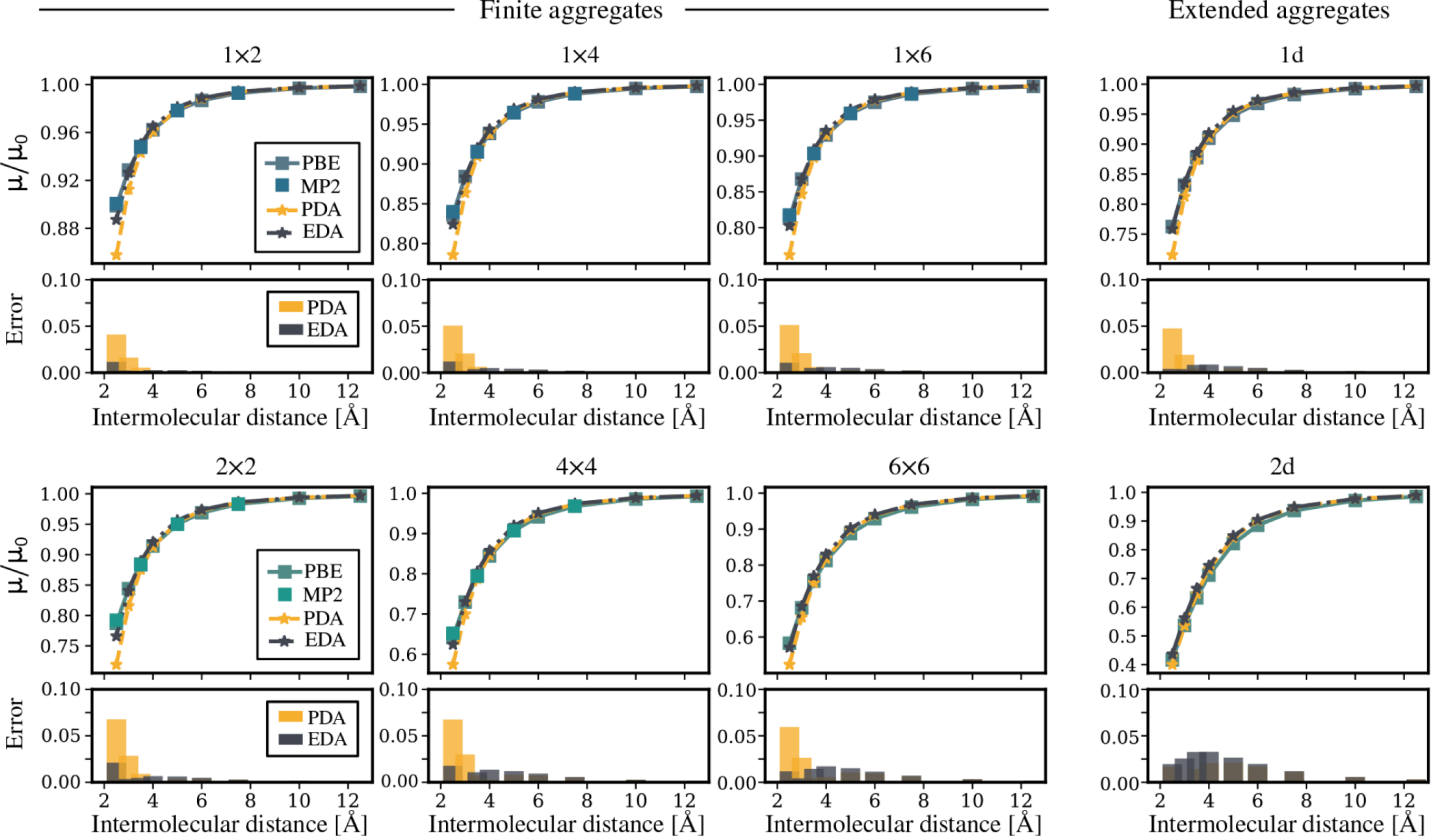
Depolarization effects in HCN aggregates. The normalized electric dipole moment, *μ*/*μ*_0_, is estimated from the two computational approaches (PBE and MP2 calculations), the point dipole approximation (PDA) and the extended dipole approximation (EDA). The first row corresponds to the normalized dipole moment of one-dimensional aggregates as a function of the intermolecular distance. In the second row, the absolute error of the two electrostatic models with respect to PBE values is represented. The third row corresponds to the normalized dipole moment of two-dimensional aggregates. In the last row, the absolute error of the classical models with respect to PBE values is represented.

For all considered HCN aggregates, PBE calculations show excellent agreement with MP2 values. For this reason, we use the PBE depolarization curve as the reference and estimate the deviation of the electrostatic models from the PBE values. As shown in Figure 1, the extended dipole approximation aligns exceptionally well with PBE data for all HCN aggregates, regardless of the size of the aggregate or the intermolecular distance. On the other hand, the point dipole approximation introduces a small deviation (below 0.1 or 10% error) at short intermolecular distances (*a* smaller than ∼ 3.5 Å). This demonstrates that even for small linear molecules, incorporating dipole size into the electrostatic model can improve the description of depolarization effects in densely packed aggregates. Finally, we notice a pattern-breaking behavior for the 2D array, where the error of the point dipole approximation is significantly reduced or even canceled.

While the primary goal of this work is not to assess the accuracy of computational approaches in describing depolarization effects in polar ligand aggregates, some relevant conclusions can be drawn from the depolarization curves of HCN aggregates, either complementing or challenging previous computational results. These curves align well with semiempirical calculations performed on finite aggregates,^9^ The reported trends of μ with respect to the number of molecules and intermolecular distance are consistent with our results for this simple linear molecule. Furthermore, we confirm the hypothesis that the dipole moment of finite clusters saturates as the number of molecules increases, explicitly demonstrating that the saturation value corresponds to that of the infinite case. For the depolarization curve of the monolayer (or 2D array), we propose that the dipole moment saturates at large intermolecular distances corresponding to low molecular densities. This contrasts with the depolarization curves reported in ref. ^10^ where the dipole moment is shown to saturate at high molecular densities. Additionally, depolarization curves estimated from different computational approaches were compared,^10^ including DFT with the PBE functional and coupled-cluster calculations, concluding that PBE strongly overestimates depolarization at low molecular coverage but performs better at high coverage due to error compensation. However, the perfect alignment of our DFT values with MP2 data contradicts their conclusion that PBE systematically overestimates depolarization effects for any molecular density of the aggregate, due to its systematic overestimation of the dipole moment and molecular polarizability of isolated molecules. To conclude, as shown in Figure 1, although PBE and MP2 calculations yield different values of μ_0_, normalizing the depolarization curves enables a more sensitive comparison between computational approaches and electrostatic models.

### Aromatic-Based Compounds

We consider finite and infinite two-dimensional aggregates of several aromatic-based compounds with intermolecular distances ranging from 6 Å to 21 Å. As illustrated in Figure 2, we start with 4-nitroaniline and then progressively increase the length of the π-conjugated system between the -NH_2_ and -NO_2_ groups. This progression results in 4-amino-β-nitrostyrene, 4-amino-4^′^-nitrobiphenyl, and 4-amino-4^′^-nitrostilbene. Consequently, we examine compounds spanning a wide range of molecular polarizabilities, from 16.37 Å^3^ to 39.1 Å^3^, and molecular sizes, from 7 Å to 14 Å (see the Supporting Information for more details).

**Figure 2:**
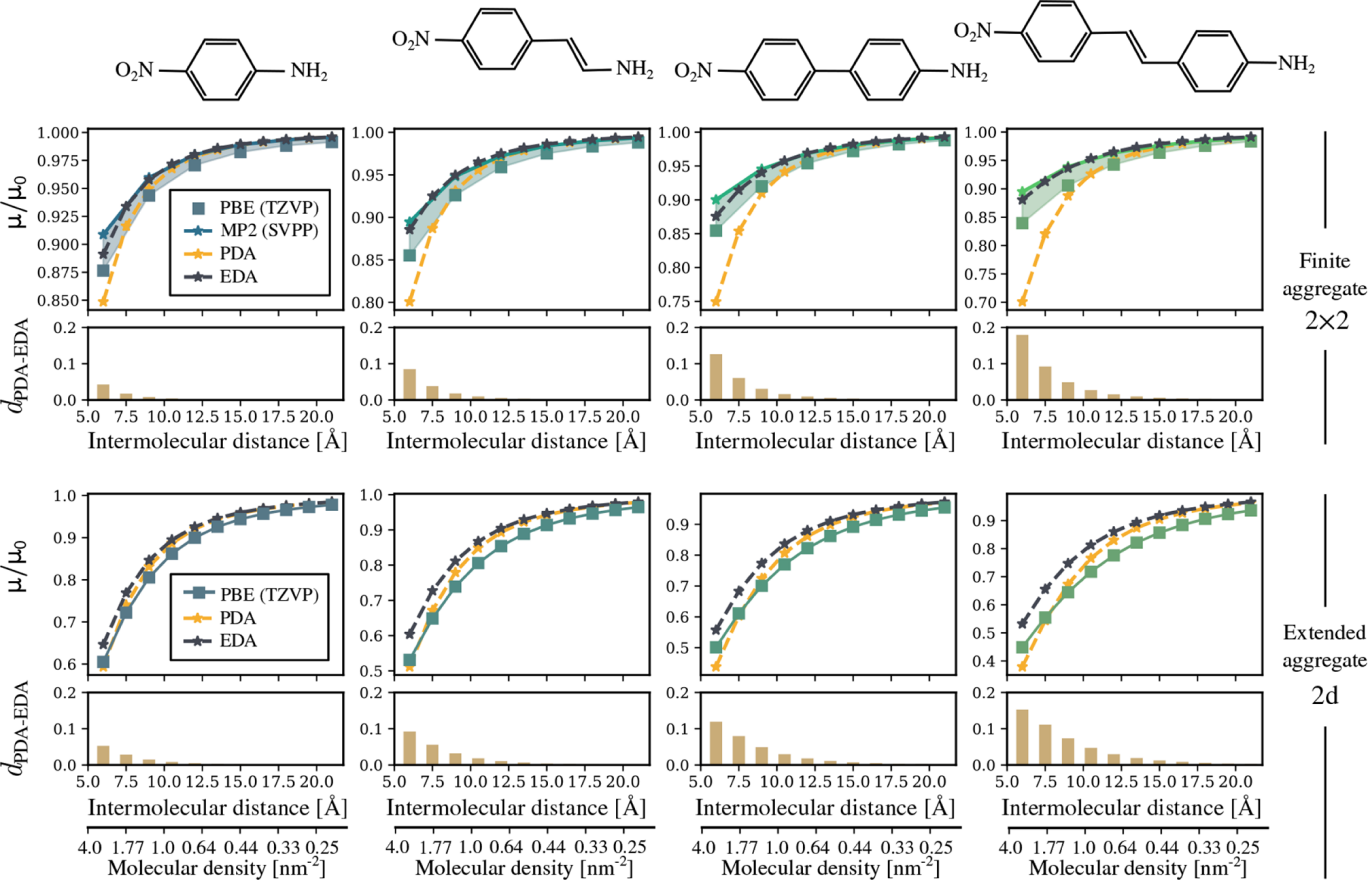
Depolarization effects in aggregates of conjugated compounds from computational approaches (PBE and MP2 calculations) and electrostatic models: point dipole approximation (PDA) and extended dipole approximation (EDA). Each column corresponds to a different aromatic-based compound. In the first row, the normalized dipole moment for the 2 × 2 aggregates as a function of the intermolecular distance is shown. PBE values are obtained with the def2-TZVP basis set, whereas MP2 values are obtained with a smaller basis set (SVPP). In the second row, the distance between both electrostatic models as a function of the intermolecular distance is represented. In the third row, the normalized dipole moment for 2D arrays is shown as a function of the intermolecular distance and/or the molecular density. In the last row, the distance between the classical models, PDA and EDA, is represented.

In Figure 2, we present depolarization curves for aromatic-based compounds obtained both from computational approaches and electrostatic models. For the finite 2 × 2 cluster, MP2 calculations were performed using the def2-SVPP basis set to reduce computational cost given the molecular sizes. These MP2 values were compared to PBE data computed with the def2-TZVP basis set. While deviations between MP2 and PBE results might *a priori* be attributed solely to the differences in basis sets, PBE­(TZVP) systematically overestimates depolarization curves relative to MP2­(TZVP) for small aromatic compounds. As shown in the Supporting Information for pyridine, aniline, chlorobenzene and bromobenzene, PBE­(TZVP) overestimates depolarization for finite 2 × 2 and 3 × 3 aggregates, whereas MP2­(SVPP) consistently underestimates it. These discrepancies can be traced back to the tendency of each approach to overestimate or underestimate the molecular polarizability of the isolated molecule (see Supporting Information). Consequently, for aromatic-based compounds, the most accurate depolarization curves lie between the upper bound given by MP2­(SVPP) and the lower bound defined by PBE­(TZVP).

Given the upper and lower bounds of the depolarization curves established from computational approaches, the extended dipole approximation outperforms the point dipole approximation for finite aggregates. It consistently falls within these bounds for all aromatic-based compound and any intermolecular distance. The discrepancy between these classical approaches becomes more pronounced as the length of the π-conjugated system between functional groups increases and as the intermolecular distance decreases. For the largest compound and the shortest intermolecular distance, the deviation between the normalized dipoles predicted by the electrostatic models approaches 20%.

For monolayers, establishing bounds for depolarization curves from computational approaches is challenging due to the lack of MP2 values. However, based on trends observed in small aromatic aggregates of sizes 2 × 2 and 3 × 3 (see Supporting Information), the discrepancy between PBE and MP2 persists as the number of nearest neighbors increases and may even slightly intensify. Regardless of PBE’s absolute accuracy in describing depolarization effects in monolayers, it can still be considered the lower bound for these effects. Indeed, as with finite aggregates, the extended dipole approximation (EDA) remains above PBE values for any compound and any intermolecular distance. For the point dipole approximation (PDA), a slight change in behavior occurs when going from finite aggregates to the monolayer, as the deviation between PBE and PDA diminishes with an increasing number of nearest neighbors. A similar trend was observed in the case of HCN aggregates, where PDA errors were significantly reduced in the 2D case. However, the difference in normalized dipoles between both models follows a similar trend when compared to finite aggregates. If the extended dipole approximation is assumed to be the reference depolarization curve, a tentative error estimate for the well-established point dipole approximation can be obtained. For instance, a ∼ 10% deviation is expected for a monolayer of 4-amino-4’-nitrostilbene with a molecular density of 2 nm^–2^.

As evident from Figure 2, PDA accurately describes depolarization effects in small aromatic monolayers, even in highly packed monolayers or those with molecular densities exceeding 1.5 nm^2^. Studies that have employed this classical model to determine the monolayer dipole formation in substituted benzene rings^8^ or to evaluate the energy contribution of image arrays in small benzene-based anchors^11^ can therefore be considered reliable. Even for aggregates composed of small donor–acceptor compounds, where a donor group (such as -NH_2_) is separated from an acceptor group (such as -NO_2_) by a benzene ring, both PDA and PBE are expected to provide reasonable depolarization curves. However, for donor–acceptor systems with π-conjugated systems longer than a benzene ring and small intermolecular distances PDA may introduce significant errors.

## Conclusions

The accuracy of the widely used point dipole approximation and the proposed extended dipole approximation in assessing depolarization effects in polar aggregates was evaluated by comparing these classical models to computational methods (PBE and MP2 calculations). The standard point dipole approximation describes depolarization solely thorough the effective molecular polarizability, while the extended dipole approximation introduces an additional parameter: the molecular size. For the simple linear molecule (HCN), the single-parameter electrostatic model provides accurate results, with errors within 5% for densely packed aggregates. However, at intermolecular distances comparable to the molecular size, incorporating a second parameter further reduces deviations, even for the simple HCN molecule. A similar trend was observed for small aromatic compounds, such as aniline and 4-nitroaniline. However, as the length of the π-conjugated system between donor and acceptor groups increases, discrepancies between classical approaches become more pronounced. In particular, the point dipole approximation shows significant deviations from computational references at short intermolecular distances.

Although establishing the accuracy of computational models in assessing depolarization effects was not the scope of the present work, a few conclusions can be drawn from our depolarization curves. By using MP2 data as reference, we showed that the PBE approximation does not systematically fail in describing depolarization curves. If the molecular dipole in the aggregate is normalized by the molecular dipole of the isolated molecule, PBE aligns well with MP2 calculations for a wide range of molecules and intermolecular densities. At short intermolecular distances, or the ones comparable to the size of the aggregated molecule, PBE overestimates depolarization effects. This trend can be attributed to the overestimation of the molecular polarizability, $\alpha_{\mathrm{iso}}$ , compared to CCSD values for the gas phase molecules.

Classical models based on one or two molecular parameters provide reference depolarization thresholds in polar-ligand aggregates. In particular, the proposed extended dipole approximation outperforms the point dipole approximation and PBE calculations for packed aggregates of compounds with π-conjugated systems longer than a benzene ring. This conceptually simple model provides reliable predictions when accurate quantum-chemical methods are computationally prohibitive. Hence, it offers an efficient prescreening tool for selecting polar-organic candidates in surface functionalization.

## Supplementary Material



## Data Availability

The data supporting the findings of this study are openly available at Zenodo^23^ via 10.5281/zenodo.15635710.

## Notes

Computational model described in detail. Molecular properties in the gas phase for all considered molecules. Additional depolarization curves for finite aggregates of small aromatic compounds (PDF)
