# Supplementary material for: Reference Depolarization Values for Polar-Organic Aggregates
Source: J Chem Theory Comput. 2025 Oct 27;21(21):11190–6. doi: 10.1021/acs.jctc.5c01055 (PMC12613312; doi:10.1021/acs.jctc.5c01055)
Supplement: Supplementary file 1 [file ct5c01055_si_001.pdf]

Supporting Information: Reference depolarization values for polar-organic aggregates

Gabriela Herrero-Saboya,[a,*] Matic Poberžnik,[b] Nicolas Salles,[a] Layla Martin-Samos[a]
[a]CNR-Istituto Officina dei Materiali (IOM), c/o SISSA, I-34136 Trieste, Italy
[b]Department of Physical and Organic Chemistry, Jožef Stefan Institute, Jamova 39, SI-1000 Ljubljana, Slovenia

S1 Computational model

Our computational model consists of isolated cubic aggregates with varying intermolecular distances, using molecules as found in the gas phase. Since both finite and infinite aggregates are considered, all calculations were performed using the Gaussian 16 package for electronic structure calculations[1]. For DFT calculations, we employed the PBE exchange-correlation functional[2]. Several basis sets were used and tested for this work (see the following section S2), including the def2-SVPP, the def2-TZVP ant the def2-TZVPP localized basis sets[3]. DFT values presented in the main text are obtained with the def2-TZVP basis set, whereas MP2 values are determined with both def2-SVPP and def2-TZVP. The choice of basis set was guided by computational resources limitations and the convergence of the electric dipole moment and polarizability of isolated molecules (section S2).

Periodic boundary conditions (PBC) were imposed to simulate infinite one-dimensional (1D) and two-dimensional (2D) arrays. Lattice cubic vectors $a\hat{\imath}$ and $a\hat{\jmath}$ were used for the 2D case. The k-point sampling was done as implemented in the Gaussian 16 package.

The gas-phase configuration of the molecules considered in this study corresponds to the optimized DFT geometry of the isolated molecule. Electric dipole moments were determined using a field-independent basis[1]. Unless otherwise stated, the reported values of the dipole polarizability are isovalues.

S2 Molecular properties in the gas phase

Since the main focus of the manuscript is to investigate depolarization effects in molecular aggregates, structural and electronic properties of the aggregated molecules in the gas phase are discussed in detail here. In particular, PBE and MP2 values for the dipole moment and polarizability are compared to CCSD values and reported experimental data.

S2.1 Hydrogen cyanide

From a standard DFT calculation (using the PBE functional), we find that the optimal distance between the hydrogen atom and the nitrogen atom is 2.23 Å. This value is in good agreement with the interatomic distance of 2.25 Å found from MP2 calculations.

For the relaxed PBE geometry, we determine the electrical dipole moment and molecular polarizability using PBE, MP2 and CCSD calculations. The results are presented in Table S1. Three different basis sets were tested and the computational values were compared to experimental data[4]. For HCN, all three computational approaches show reasonable agreement when using the two largest basis sets, def2-TZVP and TZVPP. While the MP2 method overestimate the dipole moment, the polarizability aligns closely to the CCSD value. However, when the smallest basis set, def2-SVPP, is used both μ and α are systematically and significantly underestimated compared to the values obtained with a larger basis set.

S2.2 Small aromatic molecules

We describe the structural and electronic properties of pyridine, aniline, chlorobenzene, and bromobenzene in the gas phase. While the main manuscript focuses on aniline aggregates, the supplementary materials

Basis set	Dipole moment, μ [D]				Polarizability, α [Å^3]			
	PBE	MP2	CCSD	Exp.[4]	PBE	MP2	CCSD	Exp.[4]
def2-SVPP	2.76	3.21	2.92		2.00	1.90	1.93	
def2-TZVP	2.93	3.30	3.03	2.98	2.53	2.41	2.42	2.59
def2-TZVPP	2.92	3.30	3.01		2.54	2.42	2.44	

TABLE S1: Electrical dipole moment and molecular polarizability for the HCN molecule from different computational approaches. The dipole moment is given in D, whereas the molecular polarizability is in Å^3.

discuss aggregates of the other small aromatic molecules. Our PBE calculations found optimal geometries with the following molecular sizes: 3.9 Å (pyridine), 5.81 Å (aniline), 5.62 Å (chlorobenzene) and 5.78 Å (bromobenzene). From MP2 calculations, these values are: 3.91 Å, 5.82 Å, 5.62 Å and 5.78 Å, respectively. Note that molecular size is defined as the largest difference between atomic coordinates along the axis parallel to the dipole moment.

For the relaxed PBE geometries, we determined the electric dipole moments and molecular polarizabilities of the aromatic compounds from PBE, MP2 and CCSD calculations (Table S2). Three different basis sets are tested, and computational values are compared to experimental data[4].

Molecule	Basis set	Dipole moment, μ [D]				Polarizability, α [Å^3]			
		PBE	MP2	CCSD	Exp.[4]	PBE	MP2	CCSD	Exp.[4]
	def2-SVPP	2.11	2.32	2.22		8.80	8.47	8.32	
Pyridine	def2-TZVP	2.18	2.31	2.25	2.2	10.11	9.85	9.68	9.49
	def2-TZVPP	2.17	2.31	2.24		10.18	9.93	9.76	
	def2-SVPP	2.29	1.76	2.00		11.23	10.77	10.58	
Aniline	def2-TZVP	2.11	1.74	1.89	1.5	12.9	12.47	12.24	11.58
	def2-TZVPP	2.11	1.73	1.87		13.01	12.61	12.36	
	def2-SVPP	1.43	1.88	1.61		11.35	10.79	10.61	
Chlorobenzene	def2-TZVP	1.59	1.93	1.71	1.78	13.23	12.75	12.54	11.86
	def2-TZVPP	1.58	1.92	1.69		13.3	12.84	12.62	
	def2-SVPP	1.53	1.95	1.75		12.56	11.90	11.73	
Bromobenzene	def2-TZVP	1.72	2.08	1.82	1.74	14.6	14.01	13.8	14.7
	def2-TZVPP	1.71	2.07	1.79		14.67	14.09	13.87	

TABLE S2: Electrical dipole moments and molecular polarizabilities for the aromatic molecules from different computational approaches. The dipole moment is given in D, whereas the molecular polarizability is in Å^3. Note that the iso-value of the polarizability is given for the computational values.

When comparing computational dipole moments and polarizabilities with experimental data for small aromatic compounds, the CCSD method with the largest basis set consistently provides the most accurate results. While both PBE and MP2 methods yield reasonable values compared to experimental data, MP2 aligns more closely with CCSD. Decreasing the basis set from def2-TZVPP to def2-TZVP has little impact on μ and α for the molecules considered. However, the smallest basis set leads to significant deviations from

the *converged* values.

Based on these observations, CCSD calculations are expected to provide the most accurate depolarization curves for aromatic aggregates. Due to their high computational cost, depolarization curves are obtained using the PBE and MP2 approaches with the def2-TZVP basis set. It is important to note that PBE tends to overestimate molecular polarizabilities compared to fully converged CCSD values. Moreover, MP2 calculations with the def2-TZVP basis set can become computationally demanding for larger compounds, forcing the use of the smaller def2-SVPP basis set. In this setup, α tends to be underestimated.

S2.3 Aromatic-based compounds

We finally consider aromatic-based compounds consisting of two functional groups (-NH_2 and -NO_2) separated by a π-conjugated chain. The four molecules studied are: 4-nitroaniline, 4-amino-β-nitrostyrene, 4-amino-4′-nitrobiphenyl, and 4-amino-4′-nitrostilbene. Our DFT calculations found optimal geometries with the following molecular sizes: 6.73 Å, 9.42 Å, 11.09 Å and 13.59 Å. The electric dipole moments were estimated to be 7.76 D (7.93 D), 9.93 D (9.78 D), 9.68 D (9.34 D) and 11.08 D (10.26 D), respectively, from PBE (MP2) calculations using the def2-TZVP (def2-SVPP) basis set.

Notice that in the case of 4-amino-β-nitrostyrene and 4-amino-4′-nitrostilbene, the -CH_2 group in the π-conjugated system tilts the dipole moment of the molecule. In other words, the dipole moment of these molecules is not aligned with their principal axes-defined as the axes passing through the 1 and 4 carbons of the benzene ring(s)-but is instead tilted by an angle of approximately 6°. When defining aggregates of these molecules, the individual molecules are therefore slightly tilted to maximize the dipole moment in the direction perpendicular to the aggregate. Since no experimental molecular polarizabilities were available for these compounds, reference values are taken from MP2 calculations with a def2-TZVPP basis set: 16.37 $Å^3$, 23.39 $Å^3$, 30.70 $Å^3$ and 39.15 $Å^3$.

Although our benchmark for isolated molecules includes only a limited number of computational approaches and basis sets, we emphasize that our primary goal was to describe depolarization effects in aggregates. Therefore, the computational cost of those calculations had to be considered when selecting the method and basis set. Our results are, however, compliant with previously reported benchmarks [5–9], which included a larger set of molecules, methods and basis sets. Moreover, we used the same reference values [4] to assess the accuracy of our calculations [8,9].

S3 Finite aggregates of small aromatic compounds

In this section, we provide additional depolarization curves for small aromatic compounds, complementing those presented in the main manuscript. First, we compare depolarization curves obtained using three computational approaches to establish their general tendencies. Next, we evaluate the choice of the molecular polarizability as a parameter of the electrostatic models. We finally discuss the choice of molecular size as a parameter in the extended dipole approximation.

S3.1 Depolarization effects from different computational approaches

Figure S1 shows depolarization curves for finite cubic aggregates of pyridine, aniline, chlorobenzene and bromobenzene. They were obtained from PBE calculations using the def2-TZVP basis set and MP2 calculations using both the def2-SVPP and the def2-TZVP basis sets. The reference curve is taken as MP2(TZVP). This choice is supported by the close agreement of α values for isolated molecules to those from CCSD, as shown in table S2. For all closely packed aggregates (or $a < 9$Å), PBE(TZVP) calculations overestimate the depolarization of the ligands, while MP2(SVPP) underestimates it. This behavior is consistent with the tendency of these methods to overestimate or underestimate the polarizability of molecules in the gas phase (see table S2).

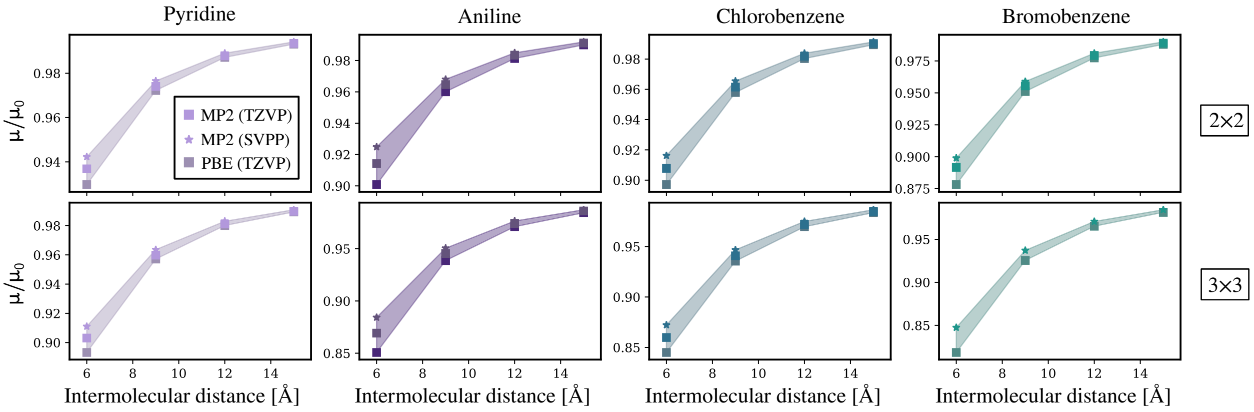

Figure S1. Depolarization curves for 2×2 and 3×3 finite aggregates of small aromatic compounds. The normalized dipole moment is represented as a function of the intermolecular distance. The curves correspond to PBE calculations using the def2-TZVP basis set, and MP2 calculations using the def2-TZVP and SVPP basis sets.

MP2(SVPP) and PBE(TZVP) depolarization curves provide upper and lower bounds, respectively, when the most accurate methods are not feasible (*e.g.* for large compounds or when including periodic boundary conditions). In other words, the *exact* depolarization curve can be assumed to lie between these two, as it is the case for MP2(TZVP) values. Although the deviations between depolarization curves in are small (less than 0.05 or 5%) in the aggregates considered, the absolute deviation of these methods from the reference curves varies depending on the molecule and the aggregate size. Establishing absolute errors of these computational approaches in describing depolarization curves is however beyond the scope of the present work. As stated in the main manuscript, computational methods are used solely as guides for comparing electrostatic models.

S3.2 Choice of the effective molecular polarizability for the electrostatic models

The normalized dipole moment in cubic aggregates, as described by the electrostatic models considered in this work, depends on an *effective* molecular polarizability, α. One plausible choice for this effective α is the isotropic value of the polarizability tensor, α_{iso}, since it represents the isotropic component of the tensor by construction. On the other hand, since the depolarization of the aggregates is analyzed in the direction parallel to the dipole moment of the aggregated molecule (taken as the z-axis), the z component of the

tensor, α_{zz}, could also be taken as the *effective* polarizability. We notice that indeed both isotropic value and the z component have been previously used as parameters of the PDA [10–12]. In the following, we test both α_{iso} and α_{zz} as effective α values in the electrostatic models. The α_{exp} is taken from [4], whereas the computational values of α are obtained using the def2-TZVPP basis set at several computational levels (Figure S2).

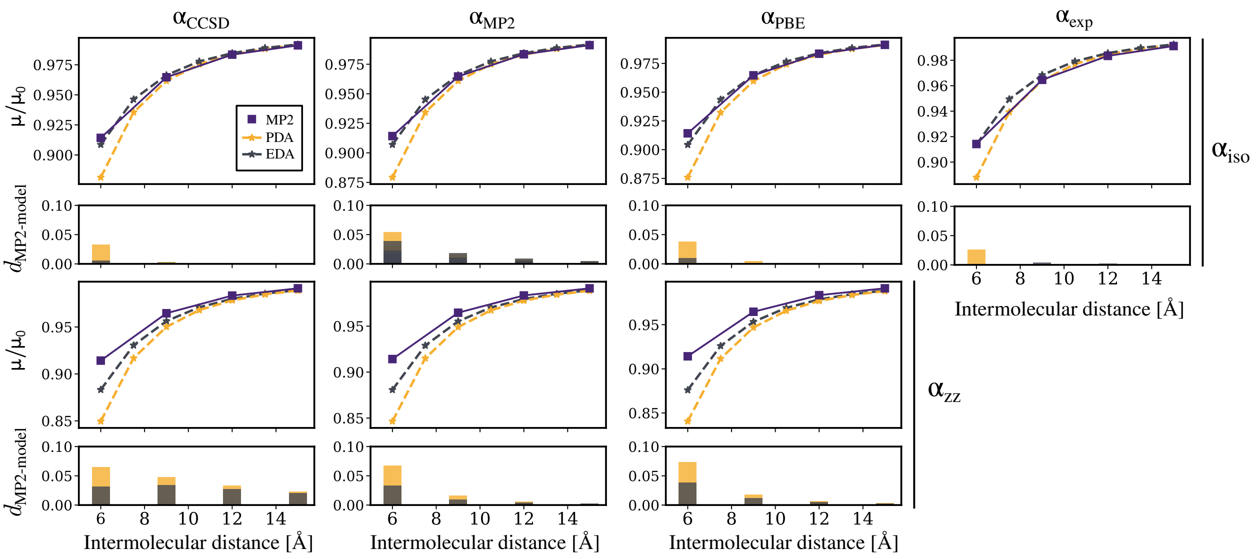

Figure S2. Depolarization curves for the aniline 2×2 finite aggregate. In the first row, the point dipole approximation (PDA) and the extended dipole approximation (EDA) using different values of α_{iso} are compared with MP2 calculations. From left to right, the CCSD estimated value, the MP2 result, the PBE value and the experimental value are employed. In the second row, the difference between both approaches is represented. In the third row, the point dipole approximation (PDA) and the extended dipole approximation (EDA) using different values of α_{zz} are compared with MP2 calculations. In the fourth row, the difference between both approaches is shown.

As shown in Figure S2, for both electrostatic models, using α_{iso} instead of α_{zz} slightly improves their accuracy compared to MP2 calculations with the def2-TZVP basis set. On a side note, when evaluating the models with α_{iso}, the depolarization curve is only slightly better when using the experimental value compared to the PBE value. In other words, the models are relatively robust to the choice of α_{iso}. Depolarization curves described by the electrostatic models in the main manuscript are obtained using α_{iso} from an MP2 calculation with the def2-TZVPP basis set, unless otherwise stated.

S3.3 Choice of dipole size in the extended dipole approximation

Besides the effective molecular polarizability, the extended dipole approximation (EDA) describes the normalized dipole in terms of the distance between point charges, d, or *the size of the dipole*. Thus, for some aggregates, the molecular distance may not be the most appropriate parameter for the model. This is the case for pyridine (see Figure S3), where EDA deviates from MP2 values by an amount comparable to PDA when d is fixed to the molecular distance of pyridine ($d = 3.9$ Å) for the shortest intermolecular distance, $a = 6$ Å. In this case, using the distance between the nitrogen atom and the opposite hydrogen atom does

not account for the electronic density localized on N, which extends beyond the benzene ring. Therefore, the extent of the electronic density should be considered when determining the d parameter. Indeed, when d is increased to 5.5 Å EDA shows better agreement with MP2 values. Note that 1.5 Å corresponds to a distance comparable to a sp^2 bond.

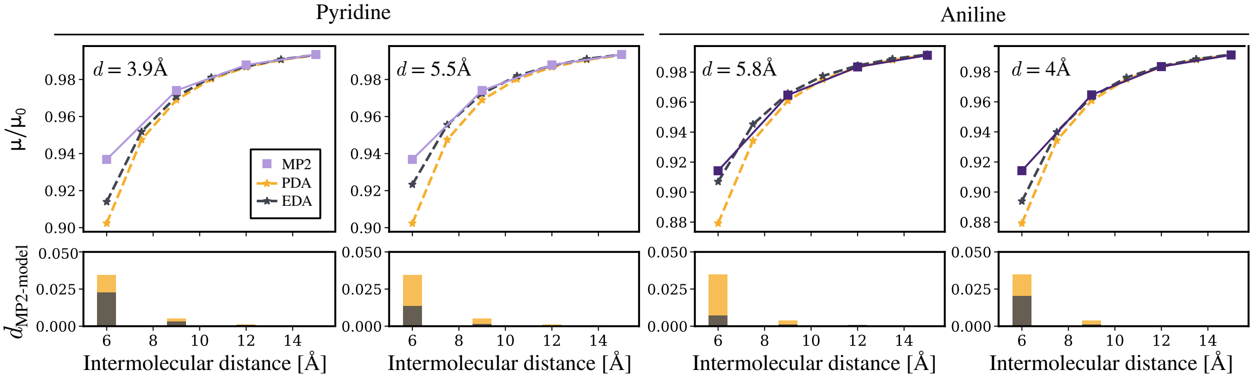

Figure S3. Depolarization curves for the pyridine and aniline 2×2 finite aggregate. In the first row, the point dipole approximation (PDA) and the extended dipole approximation (EDA) are compared with MP2 calculations, using different values of molecular distance d for the EDA model. From left to right, molecular distance of 3.9Å and 5.5Å are considered for pyridine, and distances equal to 5.8Å and 4Å are considered for aniline. In the second row, the difference between both approaches is represented.

Among the molecules considered in this work, pyridine is an exception, as taking d as the molecular distance generally yields accurate results for HCN and small aromatic compounds. For instance, the depolarization curves for aniline (Figure S3) show that EDA, implemented with the molecular size of aniline (d = 5.8 Å), provides good agreement with MP2 calculations. In Figure S3, we also present the depolarization curve described by EDA using a distance of d = 4 Å, which corresponds to the difference in atomic coordinates between the nitrogen atom and the opposite carbon in the benzene ring. This choice neglects the existence of hydrogen atoms, yet it has little impact on the accuracy of EDA when compared to MP2 values. To conclude, the parameter d in the electrostatic model is fairly robust and can generally be taken as the molecular size.

References

[1] Frisch, M. J. *et al.* “Gaussian 16 Revision C.01”, 2016 Gaussian Inc. Wallingford CT.

[2] Perdew, J. P.; Burke, K.; Ernzerhof, M. Generalized Gradient Approximation Made Simple, **1996,** *77,* 3865–3868.

[3] Weigend, F.; Ahlrichs, R. Balanced basis sets of split valence, triple zeta valence and quadruple zeta valence quality for H to Rn: Design and assessment of accuracy, *Phys. Chem. Chem. Phys.* **2005,** *7,* 3297-3305.

[4] Johnson, R. “NIST 101. Computational Chemistry Comparison and Benchmark Database”, 1999.

[5] Asger Halkier, Wim Klopper, T. H. P. J. Basis-set convergence of the molecular electric dipole moment, *J. Chem. Phys.* **1999,** *111,* 4424–4430.

[6] Bak, K. L.; Gauss, J.; Helgaker, T.; Jørgensen, P.; Olsen, J. The accuracy of molecular dipole moments in standard electronic structure calculations, *Chemical Physics Letters* **2000,** *319,* 563-568.

[7] Hickey, A. L.; Rowley, C. N. Benchmarking Quantum Chemical Methods for the Calculation of Molecular Dipole Moments and Polarizabilities, *The Journal of Physical Chemistry A* **2014,** *118,* 3678-3687 PMID: 24796376.

[8] Hait, D.; Head-Gordon, M. How Accurate Is Density Functional Theory at Predicting Dipole Moments? An Assessment Using a New Database of 200 Benchmark Values, *Journal of Chemical Theory and Computation* **2018,** *14,* 1969-1981 PMID: 29562129.

[9] Zapata, J. C.; McKemmish, L. K. Computation of Dipole Moments: A Recommendation on the Choice of the Basis Set and the Level of Theory, *The Journal of Physical Chemistry A* **2020,** *124,* 7538-7548 PMID: 32835485.

[10] Natan, A.; Zidon, Y.; Shapira, Y.; Kronik, L. Cooperative effects and dipole formation at semiconductor and self-assembled-monolayer interfaces, *Phys. Rev. B* **2006,** *73,* 193310.

[11] Piacenza, M.; D'Agostino, S.; Fabiano, E.; Della Sala, F. Ab initio depolarization in self-assembled molecular monolayers: Beyond conventional density-functional theory, *Phys. Rev. B* **2009,** *80,* 153101.

[12] Kokalj, A. Electrostatic model for treating long-range lateral interactions between polar molecules adsorbed on metal surfaces, *Phys. Rev. B* **2011,** *84,* 045418.
